# Different Long-Term Nutritional Regimens of *Drosophila melanogaster* Shape Its Microbiota and Associated Metabolic Activity in a Sex-Specific Manner

**DOI:** 10.3390/insects16020141

**Published:** 2025-02-01

**Authors:** Repac Jelena, Trajković Jelena, Rakić Marija, Lunić Tanja, Savić Tatjana, Božić Bojan, Božić Nedeljković Biljana, Sofija Pavković-Lučić

**Affiliations:** 1 Faculty of Biology, University of Belgrade, 11000 Belgrade, Serbia; marija.mandic@bio.bg.ac.rs (R.M.); tanja.lunic@bio.bg.ac.rs (L.T.); bbozic@bio.bg.ac.rs (B.B.); biljana@bio.bg.ac.rs (B.N.B.); sofija@bio.bg.ac.rs (S.P.-L.); 2 Institute for Biological Research “Siniša Stanković” National Institute of the Republic of Serbia, University of Belgrade, 11000 Belgrade, Serbia; tanjat@ibiss.bg.ac.rs

**Keywords:** *Drosophila melanogaster*, adult fly microbiota, metabolism, nutrition, sex

## Abstract

The dietary habits of fruit flies profoundly influence their fitness, morphology, and physiology yet the mechanisms underlying these effects remain incompletely understood. To address this gap, the relationship between dietary regimens and the composition and function of adult *Drosophila melanogaster* microbiota was investigated in the present study. The adult fly microbiota communities that were reared for long time on five different diets were characterized by means of 16S rRNA sequencing. Obtained results revealed distinct community structures associated with each dietary regimen, which was additionally corroborated through machine learning-based analysis. In general, sugar-rich diets correlate with microbial ecosystems of higher richness/diversity. Dominance of the phyla *Proteobacteria* and *Firmicutes* in the microbiota was confirmed irrespective of diet, with the varying proportions of the most abundant families: *Acetobacteraceae*, *Lactobacillaceae*, *Moraxellaceae*, *Bradyrhizobiaceae*, and *Leucostonocaceae*. Bacterial families of lower abundance also emerged as differentially present among the studied fly groups. Additionally, functional prediction provided initial clues into how nutrient availability might modulate the metabolic traits of adult fly microbiota in a sex-specific manner to meet host metabolic needs. Overall, the presented findings highlight the intricate interplay between diet, microbiota composition, and host phenotype in fruit flies, underscoring the importance of diet as a determinant of host-microbiota interactions.

## Simple Summary

The nutrition of fruit flies plays a very important role in various aspects of their lives. To expand the knowledge of the mechanisms underlying these processes, we analysed the microbiota of adult flies reared on different diets over a long period of time. The results show that a high-sugar diet correlates with a high diversity of the microbiota and that a different diet is associated with a different representation of bacterial families that are present in lower abundance. Furthermore, there is evidence that different diets lead to metabolic changes in the microbiota of the fruit fly in order to meet the metabolic needs of the host. The observed relationship between diet, microbiota and phenotypic traits of fruit flies is a step towards understanding the phenomenon that underlies the phrase “the gut is a second brain”.

## 1. Introduction

The gut microbiota (GM) of *Drosophila melanogaster* has attracted increasing interest in the scientific community due to its diverse role in the physiology, development and behavior of the fruit fly. Accordingly, GM gained the status of one of the key contributors to the overall fitness of its host [1]. Primarily, the communities of *Drosophila* gut microbes aid in the catabolism of nutrients, but also modulate immune response against pathogenic species, tolerance to stress, longevity, reproductive success, larval growth and development, and the transition to the adult stage [2,3].

Studies of symbiotic microbes from the laboratory-reared *Drosophila* gut have served as a valuable model system, providing insights into the broader principles of host-microbe interactions in other species as well [4,5,6,7,8]. Often, such studies have focused on the most abundant genera—*Lactobacillus* and *Acetobacter*—in gnotobiotic settings to understand better how the core community taxa set the rules for structuring the complex architecture of “healthy” microbial ecosystems in the gut [9,10,11]. Valuable and complementary to the data obtained from such cross-sectional studies are the results of a long-term longitudinal study in which natural *Drosophila* fly communities were examined under different environmental conditions. They show a strong functional interdependence between less common gut microbial constituents, underscoring the importance of a proper holistic understanding when addressing GM functionality in fruit flies [12].

In general, laboratory reared *Drosophila* flies harbor simpler GM communities (usually up to 8 different species) compared to their natural habitat counterparts (harboring up to 30 species), which mainly belong to the phyla *Proteobacteria* and *Firmicutes* [9,13], with the most common families being *Acetobacteraceae*, *Lactobacillaceae*, *Streptomycetaceae*, *Enterobacteriaceae*, *Burkholderiaceae*, *Leuconostocaceae*, and *Bradyrhizobiaceae* [12]. It is assumed that under laboratory conditions, gut microbes form transient associations with the host that are almost non-heritable, such that maintenance of the *Drosophila* GM depends almost exclusively on the stable influx of microbes from the food matrix [9,13].

How different diets affect the composition of symbiotic microbial communities of *Drosophila* has been mainly studied through quantitative variation (varying the concentration) of the main nutrients from) food matrix [14,15,16]. Contrarily, how microbiota itself affects metabolic traits and overall fitness of its host under different nutritional regimens has been more thoroughly documented in the literature [9,11,17,18,19,20]. In our previous studies, systematic evaluation of different fitness aspects (dynamics of eclosion, developmental time and egg-to-adult survival, mating behavior, reproductive success) and phenotypic traits (wing size and shape, the profile of cuticular hydrocarbons) for flies reared >450 generations on five different laboratory diets (cornmeal, apple, banana, carrot, and tomato) was performed [21,22,23,24,25]. Mating preference, reproductive success, the profile of cuticular hydrocarbons, along with the wing size and shape (important determinants of sexual attractiveness)—all emerged significantly different between flies reared on aforementioned diets.

A clear association between *Drosophila* fitness/morphology and varying nutritional regimens led to examining the influence of long-term fruit fly nutritional habits on the adult fly microbiota composition and associated metabolic potential, to detect the most likely mediator on a nutrition-behavior axis. By employing 16S rRNA amplicon sequencing, the adult fly microbiota composition for flies reared on five different laboratory diets was assessed. Downstream functional prediction provided initial clues on how nutrient availability might modulate the capacity of symbiotic microbes to complement for hosts metabolic needs. Finally, machine learning-based analysis was employed to confirm if adult fly microbiota is distinctive enough between the examined fly groups, to putatively serve as a predictor of unknown fly nutritional regimen/sex.

## 2. Materials and Methods

### 2.1. Flies, Food Substrates and DNA Isolation

Fruit flies from a natural population were reared under standard laboratory conditions for >450 generations on five different diets: standard cornmeal-sugar-yeast (S), banana (B), apple (A), tomato (T), and carrot (C). The protocol for the preparation of these diets and nutritional content was described in [26]. To avoid possible effects of genetic drift, a large population was maintained without competition. The flies were reared in twenty 250 mL glass bottles per substrate, about 2000 individuals per strain (about 100 individuals per bottle), under optimal laboratory conditions for species, i.e., at a temperature of 25 °C, a relative humidity of 60%, an illuminance of 300 lx and a 12:12 h light:dark cycle. The study groups corresponded to the combinations of fly sex and the diet of rearing, 10 in total. For each group, whole body homogenates were prepared in liquid nitrogen by using 20 frozen flies per group for DNA isolation. The genomic DNA was isolated using the Blirt EXTRACTME GENOMIC DNA KIT (Blirt, Gdańsk, Poland) according to the manufacturer instructions for isolating the insect DNA. Briefly, biological material (fly homogenates) was placed in 2 mL tubes and lysed with 375 μL GL Buffer. After vortexing and centrifugation, 25 μL Proteinase K was added, followed by incubation at 55 °C. Next, 4 μL RNase A was added and incubated at 37 °C. Subsequently, 400 μL GB Buffer was added, and the mixture was centrifuged. The supernatant was transferred to a DNA Purification Column, and after centrifugation, wash steps were performed using GW1 and GW2 Buffers. The column was then transferred to a new tube, and DNA was eluted with Elution Buffer. For each group, technical triplicates were made. Purified DNA was stored at −20 °C for subsequent use. Quality control of isolated DNA was evaluated by 1% agarose gel electrophoresis.

### 2.2. DNA Sequencing and Downstream Analysis

The 16S rRNA gene amplicon sequencing was performed on an Illumina paired-end platform. PCR amplification of isolated genomic DNA was performed using barcoded primers targeting the hypervariable V3-V4 region of the 16S rRNA gene: 341F (5′-CCT AYG GGR BGC ASC AG-3′) and 805R (5′-GGA CTA CNN GGG TAT CTA AT′), producing 1,919,718 reads in total for 30 samples (5 diets × 2 sexes × 3 replicas), with an average of 63,990.6 reads per sample. The downstream data analysis was performed by using the Qiime2 pipeline, version 2022.8 [27] for raw data processing, diversity metrics calculation, taxonomy binning and machine learning analysis and stand-alone version of the MicFunPred program (https://github.com/microDM/MicFunPred, accessed on 23 January 2025) [28] for predicting the metabolic potential of inferred adult fly microbial communities.

Raw data processing consisted of reads quality filtering and trimming to the length of 400 base pairs, chimera removal and denoising with the DADA2 algorithm [29] to produce feature table with ASV sequences. Low frequency features (relative abundance below 0.01%) were filtered out. Prior to calculating the diversity metrics, sampling depth was set to 35,000 (rarefaction cut off), according to the number of features present in the sample with the lowest number of features. Alpha diversity was inferred by calculating the Shannon diversity index, while beta diversity was inferred by calculating the phylogenetically informed metric weighted UniFrac. Obtained UniFrac distances were used for constructing the Bray-Curtis emperor plot. In both cases, pairwise comparisons between all the possible combinations were performed, depending on the invoked metadata category (Diet, Sex, and the Diet-Sex combination). Taxonomy binning was performed by using the naïve Bayes classifier, trained on the V3-V4 16S rRNA gene segment, against the SILVA138 database of 16S rRNA gene sequences [30], at the 99% cut off. After inspection, contaminant taxa (corresponding to sequences of eukaryotic origin—mitochondria/chloroplast/Diptera, sequences without taxonomic assignment at the phylum level and maritime bacterial taxa classified to orders *Gammaproteobacteria* Incertae Sedis and *Alphaproteobacteria* Clade I) were filtered out, so were the two samples with high abundance of contaminants (samples M_9.3 and M_10.1 corresponding to the microbiota of adult male and female flies reared on the A diet, respectively). Analysis of differentially abundant taxa was assessed through the ANCOM (Analysis of Composition of Microbiomes) test by invoking the same metadata categories as for the diversity analysis. Machine learning was performed by using the qiime2 plugin sample-classifier with default parameters, which calls the random forest algorithm to perform classification according to the chosen metadata category (Diet and Sex). Finally, for inferred microbial communities, the stand-alone version of the machine learning-based MicFunPred program, which operates on normalized ASV abundance data, was used with default parameters to predict functional attributes of examined microbial communities.

## 3. Results

To evaluate the influence of different long-term standardized nutritional regimens on *D. melanogaster* microbiota composition was performed, 16S rRNA amplicon sequencing of the DNA isolated from whole tissue homogenates. In total, 1,919,718 reads were obtained after sequencing, with the mean value per sample equaling 63,990.6 reads. The sequencing quality was high, as evidenced by the low sequencing coverage span across the samples (minimal number of obtained reads per sample equals 54,684, while the maximal value corresponds to 78,144 sequencing reads in a sample). Also, consistently high number of quality filtered reads (minimum 88.39% and maximum 93.93%) and non-chimeric reads (minimum 60.53% and maximum 91.87%) across samples was observed. In terms of the non-chimeric reads metric across different samples, predominantly uniform distribution was observed, with the exception of samples obtained from male flies reared on the B diet (lower values obtained for all three samples spanning from 64.41% to 65.5%) and samples obtained from female flies reared on the S diet (higher values obtained for all three samples spanning from 89.86% to 90.61%). The distribution of filtered high-quality reads was also uniform, with the exception corresponding to samples obtained from male flies reared on the T diet that clustered within the lowest values (from 88.39% to 88.7%). The denoising process produced a feature table comprising 7036 unique ASV sequences with the total frequency of 1,515,804 counts, distributed from 40,536 up to 66,127 counts across different samples. After filtering out low frequency features (<1% of total ASV counts), 381 unique ASV sequences with the total frequency of 1,420,205 counts remained, distributed from 36,597 up to 62,969 counts across different samples. Finally, after taxonomic annotation, two outlier samples with pronounced contamination (one obtained from male, and the other from female flies reared on the A diet, respectively) were also filtered out.

**Figure 1:**
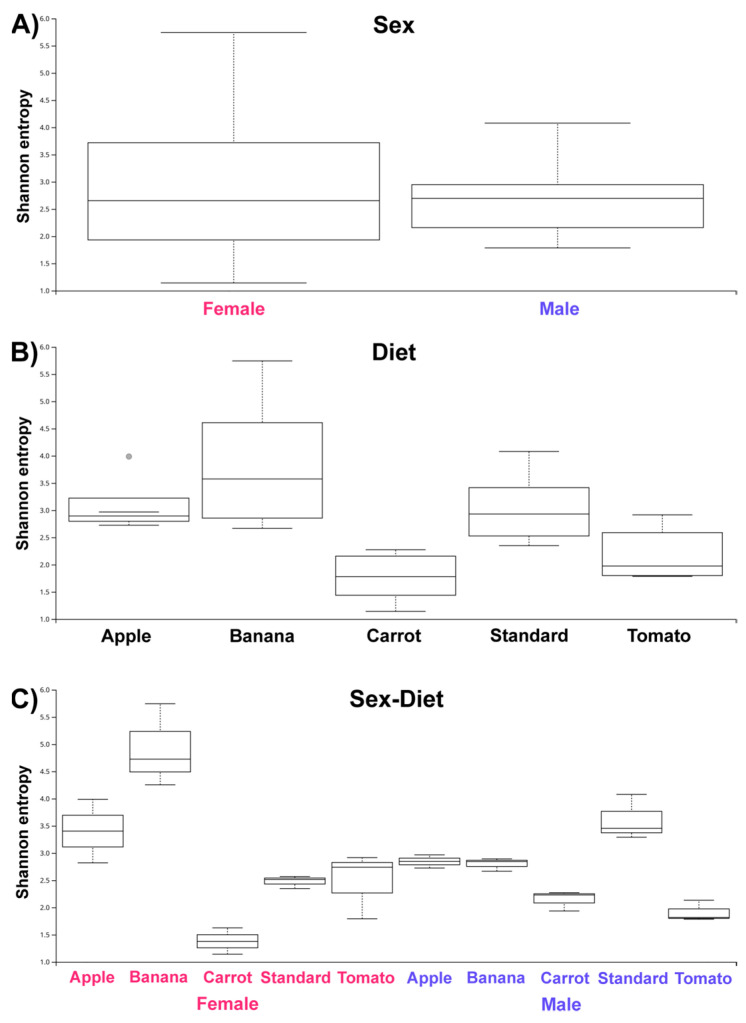
Boxplots showing the Shannon diversity index for microbial communities of flies: (**A**) for samples grouped according to the fly sex; (**B**) according to rearing diets; and (**C**) according the both fly sex and the rearing diet.

To infer the overall structure and associated differences of examined microbial communities, metrics of alpha and beta diversity were calculated for all the sample metadata categories (samples grouped according to the Sex (2 groups), Diet (5 groups), and the combination Sex/Diet (10 groups)). For alpha diversity, the Shannon diversity index was chosen, as it provides information on both the richness (number of different features, i.e., microbial taxa) and evenness (the distribution of different feature counts in the samples) for interrogated samples. When comparing symbiotic microbial communities of male and female flies, regardless of the nutritional regimen, the similar mean value for alpha diversity was observed (H = 0.008; *p* > 0.05), although with higher standard deviation for samples obtained from female flies (Figure 1A). When comparing the same communities of flies reared on different diets, regardless of sex, more pronounced differences emerge (Figure 1B). The diets B, S, and A, characterized by the high carbohydrates to protein ratio, coincide with adult fly microbiota of higher mean alpha diversity. Among these, the B diet coincides with both the highest mean value and standard deviation of calculated Shannon diversity index. Diets C and T coincide with microbiota of similar, and overall lower, mean values for Shannon diversity index. In terms of statistical significance, the comparison of microbial communities across all the groups produces *p*-value of 0.002, while for pairwise comparisons (SI, Excel file: alpha-diversity-kruskal-wallis-pairwise-Diet), statistically significant differences were detected between the following diets: A and C (H = 6.545; *p* = 0.01), B and C (H = 8.308; *p* = 0.004), B and T (H = 5.026; *p* = 0.02), C and S (H = 8.308; *p* = 0.004), and also borderline (*p* = 0.05) statistically significant difference for the pairs of samples A and T, and S and T. When considering the combination of fly sex and nutritional regimen (Figure 1C), pronounced differences between the microbial communities from different groups in terms of the Shannon diversity index remain, with the *p*-value for comparisons across all the groups equaling 0.004. In contrast, pairwise comparisons produce only borderline statistical significance between given groups (*p* = 0.05, SI, Excel file: alpha-diversity-kruskal-wallis-pairwise-Sex-Diet), this being a consequence of the smaller number of samples in individual pairwise comparisons. In general, the coincidence of diets with higher sugar content with microbiota of higher alpha diversity persists, and is more pronounced in female flies (A, B, and S diets vs. T and C diets). Finally, the comparison of microbiota obtained from flies of different sex, but reared on the same sugar-saturated diet, reveals higher diversity among communities in female flies reared on the A, and B diet, in distinction to flies reared on the S diet.

The original data presented in the study are openly available in National Center for Biotechnology Information (NSBI) repository. Accession numbers and URL are available in Supplementary Material.

**Figure 2:**
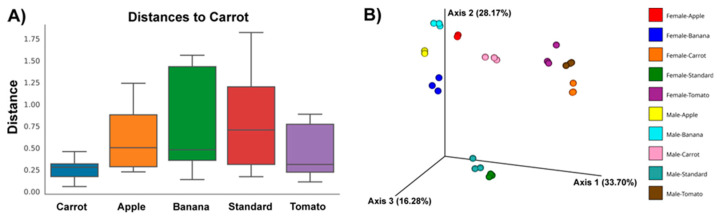
(**A**) Pairwise comparison of weighed Unifrac distance indices for samples grouped according to nutritional regimens and expressed with respect to the microbiota of flies reared on the C diet; (**B**) Bray-Curtis emperor plot of weighed Unifrac distance indices for samples grouped according to both the fly sex and rearing diet.

Mutual differences in the composition of adult fly microbiota across examined groups were next evaluated through the phylogenetically informed beta diversity metric, weighted Unifrac. Grouping the samples only according to fly sex produces statistically insignificant difference. Contrarily, grouping according to fly diets produces a statistically significant difference for beta diversity indices, when comparison was performed across all the groups (*p* = 0.05). In Figure 2A, the Unifrac distance was shown for microbial communities obtained from flies reared on different diets (with respect to microbiota of flies reared on the C diet that emerged the most distinct to other communities in terms of both alpha and beta diversity). A statistically significant difference was observed between the microbiota of flies reared on the C and B (*p* = 0.005), C and S (*p* = 0.02), and C and T diet (*p* = 0.02) for pairwise comparisons (SI, Excel file: beta-diversity-weighted-unifrac-Diet-significance_permanova-pairwise). The same trend with more pronounced statistical significance (*p* = 0.01) emerged after grouping the samples according to both fly sex and diet; however, pairwise comparisons produced no significant differences because of lower number of samples for comparison (SI, Excel file: beta-diversity-weighted-unifrac-Sex-Diet-significance_permanova-pairwise). Overall, microbial ecosystems from female flies reared on the C and S diet, and male flies reared on the S diet differ most with respect to other examined communities, as observed on the Bray-Curtis emperor plot (Figure 2B). This plot also shows clustering of microbial communities according to diet used by the flies, with additional segregation according to fly sex. Only microbiota of male and female flies reared on the C diet do not cluster together.

**Figure 3:**
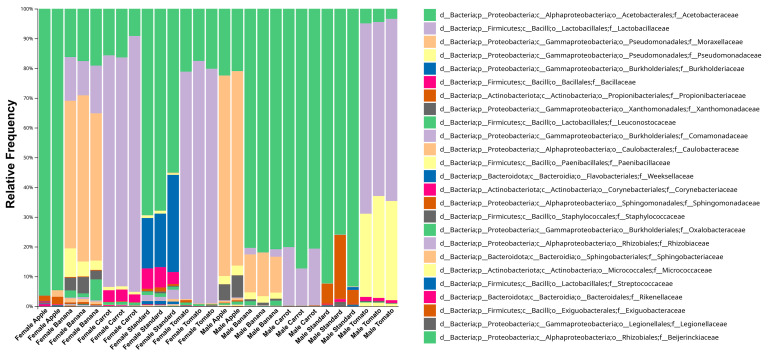
Taxabarplot showing relative frequency of annotated, filtered taxa at the family level for samples grouped according the sample metadata categories Sex and Diet.

To contextualize biologically obtained results, taxonomy binning was performed next. In Figure 3 taxabarplot was shown (level family providing full taxonomic assignment scope), with individual samples aggregated and annotated to different groups according to fly sex and the rearing diet. The number of annotated taxa corresponds to diversity established across other studies in laboratory-reared *Drosophila* flies, so as the dominance of phyla *Proteobacteria* and *Firmicutes* [9,13], with the rare occurrence of *Actinobacteriota* and *Bacteroidota* (data available on request). From the plot, a consistent share of the same taxa is easily observed for mutually grouped samples. The three most abundant bacterial families are *Acetobacteraceae*, *Lactobacillaceae*, and *Moraxellaceae*, where the two aforementioned show inverse trend of occurrence between the microbial communities of different Sex/Diet groups. Microbiota of female flies reared on the A diet is almost exclusively dominated by the family *Acetobacteraceae*, while the microbiota of male flies reared on the same diet is dominated by the *Moraxellaceae* family, followed by *Acetobacteraceae*. A substantially high share of the *Moraxellaceae* family is also characteristic for microbiota of female flies reared on the B diet, while the same diet of male fly microbiota harbors a lower share of this taxon at the expense of the dominant *Acetobacteraceae* family. Notably, both A and B diets are characterized by easily accessible sugars in high amounts. The ultimate sugar-saturated diet, S, also favors microbial communities dominated by *Acetobacteraceae* for both fly sexes; however, in female flies the next most abundant family is *Burkholderiaceae* while in males it is *Propionibacteriaceae*. The *Acetobacteraceae* family also dominates in the microbiota of male flies from the C diet, in contrast to the microbiota of the same diet of female flies that is dominated by *Lactobacillaceae*, just like the microbiota of flies reared on the T diet. Among the latter, *Lactobacillaceae* are more abundant across female flies, while the microbiota of male flies harbors a greater share of bacteria belonging to the *Pseudomonadaceae* family.

To corroborate further the patterns of different taxa occurrence across examined microbial communities, ANCOM differential abundance testing was performed next (SI, Excel files: ancom-percent-abundances-Sex, ancom-W-scores-Sex/ancom-percent-abundances- Diet, ancom-W-scores-Diet/ancom-percent-abundances-Sex-Diet, ancom-W-scores-Sex-Diet). Grouping the samples according to the fly sex reveals only weak indications of differential abundance for families *Oxalobacteraceae*, *Caulobacteraceae*, and *Rhizobiaceae*. Interestingly, these families correspond to taxa of very low abundance, which, however, mostly coexist across the samples of the same groups (microbiota of female flies from the B diet, microbiota of male flies from the A diet, and microbiota of female flies from the S diet—these all correspond to food matrices with high sugar content). Notably, *Oxalobacteraceae* are more abundant in microbiota of male flies, while *Caulobacteraceae* and *Rhizobiaceae* are more distinctive of female fly microbiota. When the grouping is performed according to the diet, more pronounced differences emerge (higher W scores), although for similar number of taxa (families *Lactobacillaceae*, *Moraxellaceae*, *Burkholderiaceae*, and *Leuconostocaceae*). In distinction to taxa targeted as differentially abundant with respect to the fly sex, these families are among the most highly abundant (the first three families are among the top five most abundant taxa, while the last one is among the top 10 most abundant taxa). *Lactobacillaceae* are highly abundant in the microbiota of flies from the B, C, and T diet; *Moraxellaceae* in the microbiota of flies from the A and B diet; *Burkholderiaceae* in the microbiota of flies from the S diet; while *Leuconostocaceae* in the microbiota from flies reared on the B and S, and to a lesser extent C and T diets. Lastly, when clustering the samples according to both metadata categories (Sex/Diet), high W scores (>20) are obtained for >20 different bacterial families, corresponding to taxa of high, medium and low abundance.

**Figure 4:**
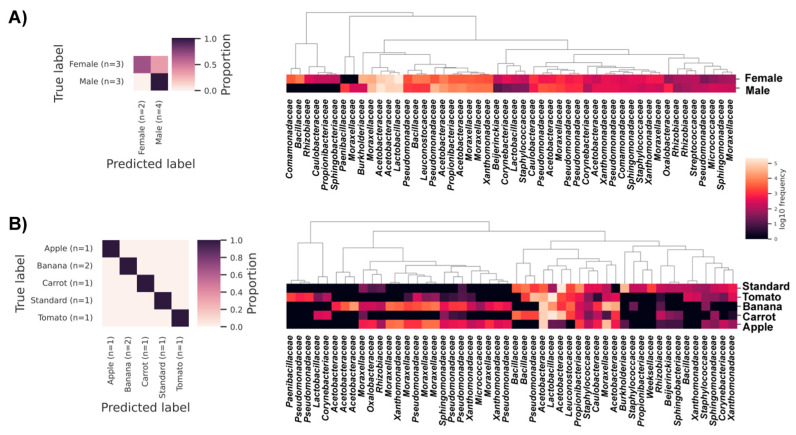
Random forest results in the form of heatmaps (on the left) showing predictive performance of symbiotic microbial communities for metadata categories Sex (**A**) and Diet (**B**). Heatmaps to the left show the predictive performance score, while the heatmaps to the right show correlation between the abundance of selected features and values of interrogated metadata category.

The optimization of statistical tests for compositional data, characteristic of microbiota studies, especially for comparing multiple samples, is still an open issue. Therefore, to corroborate ANCOM findings, a complementary, machine learning (ML) approach based on the random forest algorithm was next employed to search for distinctive features/taxa that could potentially be used as predictors for associated metadata category value. In terms of Diet, microbiota composition showed maximum predictive performance (Figure 4B, heatmap on the left), while for fly sex, somewhat lower predictive performance was observed (Figure 4A, heatmap on the left). Heatmaps to the right on the same Figure show how the abundance of most distinctive features for each interrogated metadata category correlate with their values. These features correspond to unique ASV sequences, which were shown under their taxonomic labels at the family level. The fact that distinctive features emerged among ASV sequences, belonging to families across a rang of abundance, might indicate important roles of bacteria beyond the core community for maintenance of symbiotic microbial ecosystems in *Drosophila*.

**Figure 5:**
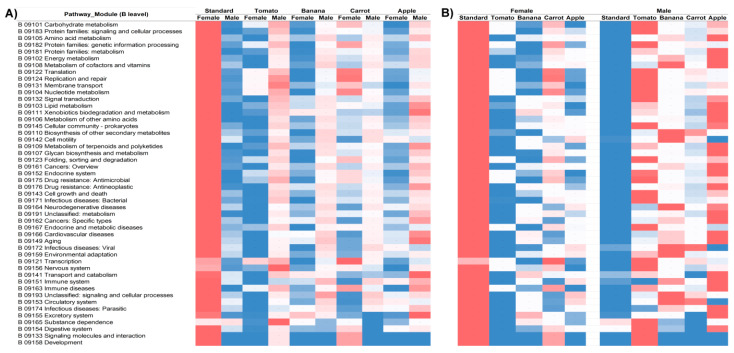
Heatmaps for top 48 most abundant KEGG level B processes predicted by MicFunPred calculated for microbial communities of flies reared on different diets for both sexes (**A**) and female/male fly sex independently (**B**). Color scale: the red indicates higher and blue lower predicted abundance for a given process.

Taxonomic profiling does not provide insight into the functionality of the examined microbial ecosystem; this can partly be gained by predicting the metabolic potential of the community. Accordingly, MicFunPred functional prediction was performed on the abundance data from the feature table and the results are shown in Figure 5 for the KEGG (Kyoto Encyclopedia of Genes and Genomes) output, hierarchical level B (the first subdivision within broader categories, for the top 48 most abundant processes). The heatmap was constructed for each metabolic process (row) individually across different metadata categories (Diet for both fly sexes on the Figure 5A, and Diet/Sex combination in Figure 5B), as these values cannot be scaled against one another for fundamentally distinct processes. Interestingly, a regular pattern spanning across virtually all the processes shown emerged. Namely, the predicted metabolic potential of microbial communities of female flies from the S diet is very high while the opposite holds for the same diet communities of male flies. The opposite trend is observed for microbial communities of flies from the diets A, B, and T, where the lower metabolic potential of female fly symbionts is compensated by the male counterparts from the same diet. The only exception to this pattern are microbial communities from the flies reared on the C diet, which in terms of beta diversity also appear the most distinct to all other groups, and differ between sexes more than any other same-diet community pairs. Consistently, on the Bray-Curtis plot, the overlap of C diet microbes of male flies with the microbes from flies (both males and females) reared on the S diet can be observed along the plane determined by the Axes 1 and 3 (Figure 2B), which aligns with the absence of the metabolic pattern opposite to the S diet-obtained, seen in all other diet categories.

## 4. Discussion

In their natural habitats, fruit flies generally feed on a variety of decaying organic substrates, such as those found in fermenting fruits and vegetables, decaying plant matter and compost, which provide the flies with carbohydrates, proteins, lipids, vitamins and minerals in varying proportions. In addition to nutrients, fruit flies also ingest various microorganisms that colonize their gut and ultimately contribute to the digestion of complex substrates, vitamin synthesis and local immune responses, juvenile growth, and reproductive behavior [1,3,9,19,31,32]. How dietary choice influences phenotypic traits and overall fitness of fruit flies has been meticulously studied for decades [33,34,35,36]. How diet—both as a source of prebiotics and of microbes themselves—shapes the ecosystems of symbiotic microbes of adult fruit fly has been less addressed. Therefore, the present study links the microbial composition and associated metabolic potential of fruit flies and their long-term diets, previously reported as strong modulators of the fly’s fitness [21,22,23,24,25].

The long-term laboratory rearing of flies with nutrient-standardized feed minimizes the influence of genetic drift and environmental factors on microbiota ecology, so the observed compositional variance can mostly be attributed to dietary choice which probably shapes communities in both intestinal and extraintestinal compartments, such as the cuticular/gland/hemolymph microbiota, and the reproductive microbiota of female flies (ovaries and oviducts)—already described in different insect species [37,38,39]. Among these, gut microbiota represents the richest microbial community symbiotically associated with fruit flies which might explain the substantial reduction in the number of unique ASVs after filtering out the low-abundance features, since part of the filtered out features probably corresponds to the steady influx of transient microbial species that fail as successful gut colonizers [10]. This suggests that the structuring of the fruit fly microbial ecosystems might not be stochastic even for laboratory-reared species as previously assumed [9].

Aside from low-abundance ASVs, two samples were also discarded due to obvious contamination coupled with a lower percentage of non-chimeric reads. Both belong to flies reared on A diet, producing lower standard deviation of the alpha diversity index in this group; however, with the mean value corresponding to the trend of high-sugar diets producing more diverse microbial communities. Consistently, only pairwise comparisons of alpha diversity indices between diets with inverse carbohydrate-to-protein ratios revealed statistically significant differences (A, B and S vs. C and T diets). Since in many aspects of fitness and behavior, diets with lower carbohydrate content produce better performing fruit flies [21], the microbiota diversity is not positively related to the underlying symbiotic value if associated microbes function as the predominant link between the fly diet and behavior. In sugar-rich diets the ready accessibility of carbohydrates probably aids the growth of both transient microbial taxa and the stable colonizers, so the ecosystem reaches the balanced state with more difficulty. This interpretation, however, might not hold in context of stressful environmental conditions, when exactly sugar-rich diets provide better tolerance to different stressors like cold and starvation [16]. Next, when the fly sex is also taken into account, it becomes evident that female flies reared on diets A and B and male flies reared on diet S produce most of the observed alpha diversity so the observed inversion might be related to more pronounced yeast fermentation in the gut of S-reared flies. How much of this effect is related to gut or the extraintestinal microbiota, functionally related to reproductive behavior, remains to be further investigated.

Consistent with alpha diversity results, calculated beta diversity indices favor the diet over the fly sex for its influence on studied microbial ecosystems and observed compositional differences. A novel finding is that the C diet produces significantly different communities both from those arising from sugar-rich (S, B) and more protein-rich food matrices (T), and also the fact that for this nutritional regimen the fly sex is equally distinctive as are the remaining diets. In contrast to typical fruit fly diets containing apple (in here the A diet) or bananas (in here the B diet), carrots provide a distinct spectrum of important nutrients, like dietary fibers, vitamins (vitamin A, vitamin C), essential minerals (very rich in K, Mg), and strong antioxidants (beta carotene, polyphenols) which obviously differentially affect the symbiotic communities of flies in a sex-specific manner (the prevalence of *Lactobacillaceae* and *Acetobacteraceae* in GM of female vs. male flies) with long-reaching effects on fitness, at least during larval stage [21]. Interestingly, *Lactobacillaceae* species it have been shown to mediate the diet-inducing mating preference in *Drosophila* flies [31], which could be the reason for such marked differential abundance between microbiota of male and female C-reared flies.

A slightly lower annotation value (proportion of unassigned taxa at the genus level) found across different samples is probably due to the less frequent study of natural microbial communities of flies, at the expense of experimentation with germ-free flies colonized with the major gut microbial community taxa, as gnotobiotic conditions allow for a very controlled experimental design that simultaneously lacks ecological perspective and knowledge of the less abundant taxa [40]. However, taxonomic analysis at the family level provided sufficient resolution of the consistency of compositional patterns when samples are grouped by sex and diet of the flies. The observed richness is consistent with diversity metrics observed in other studies using laboratory-reared *Drosophila* flies, as is the dominance of the phyla *Proteobacteria* and *Firmicutes* and the high abundance of the families *Acetobacteraceae* (most widely distributed across different groups), *Lactobacillaceae*, *Moraxellaceae*, *Bradyrhizobiaceae*, *Leucostonocaceae* [9,12,13].

High-sugar diets such as B and A favor the growth of members of the *Acetobacteraceae* and *Moraxellaceae* families, with sex-specific mutual contribution, possibly due to the influence of the reproductive microbiota. In sugar-rich S diets, *Acetobacteraceae* is also the most abundant, followed by *Burkholderiaceae* in female flies and *Propionibacteriaceae* in male flies. Members of the *Burkholderiaceae*, generally not considered ubiquitous for the fruit fly microbial communities [12], were also found to be differentially abundant in the ANCOM test, metabolize sugars under both aerobic and anaerobic conditions, so are likely complementary with yeasts present in the S food matrix during carbohydrates metabolism, nutrient recycling and cross-feeding interactions. In some other insects, for *Burkholderia* species the ability for crossing the epithelial barrier and priming of systemic immunity was shown, shedding light on important non-nutritional roles for this bacterial taxon [41]. Metabolic complementarity with yeast species can be more assumed for the *Propionibacteriaceae* family, skilled in the fermentation of sugars, utilization of lactate/succinate/ethanol for the production of short-chain fatty acids (especially of yeast-produced ethanol as a carbon source for propionate production), but also in the synthesis of B vitamins, biofilm production and a marked tolerance to acidic conditions [42,43]. Conversely, *Moraxellaceae* bacteria typical for GM of A- and B-reared flies, readily utilize various complex organic substrates; however, almost exclusively under aerobic conditions.

Further on, differential abundance testing prompted the families *Oxalobacteraceae*, *Caulobacteraceae* and *Rhizobiaceae* as putatively different for different sex fly microbiota. In low-abundance, these taxa coexist in samples correlated to sugar-rich diets, which justifies the modest W score in the ANCOM test. For these families functional redundancy could be assumed due to the propensity of their members for nitrogen fixation and recycling which could be beneficial under nitrogen-poor (low protein share) or high-fiber diet (rich in complex carbohydrates and plant polysaccharides) both for the ecosystem maintenance and for compensating the hosts metabolic needs [44,45]. In contrast to fly sex, differentially abundant families between the samples grouped according to the fly diet are the most highly prevalent microbial taxa which probably points to diet as a driving ecological force in shaping the microbial landscape in *Drosophila*. The joint inclusion of both metadata categories (Sex/Diet) marks as differentially abundant a wide array of taxa irrespective of their abundance. This is additionally confirmed with machine learning since ASVs from families across a broad abundance spectrum emerge as highly-predictive features, underscoring almost equal contribution of unequally abundant bacteria for the maintenance and functioning of adult fly microbial communities [12].

The lower predictive power of random forest algorithm for classifying samples according to the fly sex over rearing diet is nicely corroborated with the results of metabolic potential prediction, where an inverse trend of metabolic capacity/complementation is seen for the microbial communities of male and female flies reared on S vs. flies reared on A, B, and T diets over the entire spectrum of examined metabolic processes. Again, the most probable explanation is the influence of a more pronounced fermentation in the gut environment of S-reared flies, which obviously strongly modulates the possibility for coexistence between bacteria and non-bacterial members of the community [12] and is also consistent with the results from literature showing that the yeast load in the food matrix is a detrimental factor for gut microbiota-related nutritional effects on the metabolism and overall fitness of flies [16]. Noteworthy, the trend is consistent to both the inverse values of alpha diversity indices of male and female flies microbiota for sugar-rich diets (S vs. A and B) and also the fact that the S-reared microbial community is most distant to communities reared on other diets at the Bray-Curtis plot (indicator of the beta diversity). Accordingly, the only exception from observed trend is the microbiota of both male and female flies reared on C diet, which also appeared peculiar with respect to microbial communities from flies reared on other diets. What remains to be evaluated is whether yeast addition to food matrices other than S would reverse this trend and shed some more light on the underlying role of fly sex, as literature data indeed indicate that the microbiota differentially shapes metabolic output of fruit flies depending on both the diet and the fly sex [17]. Observed marked inversion of this microbiota-induced sex-specific trend certainly implies involvement of some metabolic compensation between flies of different sex, which might be important during mating and related behaviors, like the production of pheromones in male flies or perceiving/processing of sensory information in female flies, as shown previously for the influence of bacterial symbionts of *Drosophila* and fly olfactory behavior [32].

## 5. Conclusions

In summary, sugar-rich diets leading to higher microbial richness in fruit flies, does not necessarily indicate superior community performance. The dominance of *Proteobacteria* and *Firmicutes* phyla was consistent across different nutritional regimens, with varying proportions of abundant families such as *Acetobacteraceae*, *Lactobacillaceae*, *Moraxellaceae*, *Bradyrhizobiaceae*, and *Leuconostocaceae*. Less abundant bacterial families also showed differential presence among fly groups, additionally highlighting distinctiveness between them. Functional prediction revealed insights into how nutrient availability might influence the metabolic potential of the adult fly microbiota, emphasizing fermentation as important factor for shaping community functionality. Overall, obtained findings underscore the complex relationship between diet, microbiota composition, and host phenotype in fruit flies, emphasizing the role of diet in host-microbiota interactions.

## Data Availability

The sequence data generated in this study can be accessed at NCBI SRA database, under following accession numbers: SAMN46288862, SAMN46288863, SAMN46288864, SAMN46288865, SAMN46288866, SAMN46288867, SAMN46288868, SAMN46288869, SAMN46288870, SAMN46288871, SAMN46288872, SAMN46288873, SAMN46288874, SAMN46288875, SAMN46288876, SAMN46288877, SAMN46288878, SAMN46288879, SAMN46288880, SAMN46288881, SAMN46288882, SAMN46288883, SAMN46288884, SAMN46288885, SAMN46288886, SAMN46288887, SAMN46288888, SAMN46288889.

## Supplementary Materials

The following supporting information can be downloaded at: https://www.mdpi.com/article/10.3390/insects16020141/s1, Table S1: alpha-diversity-kruskal-wallis-pairwise-Diet; Table S2: alpha-diversity-kruskal-wallis-pairwise-Sex; Table S3: alpha-diversity-kruskal-wallis-pairwise-Sex-Diet; Table S4: ancom-percent-abundances-Diet; Table S5: ancom-percent-abundances-Sex; Table S6: ancom-percent-abundances-Sex-Diet; Table S7: ancom-W-scores-Diet; Table S8: ancom-W-scores-Sex; Table S9: ancom-W-scores-Sex-Diet; Table S10: beta-diversity-weighted-unifrac-Diet-significance_permanova-pairwise; Table S11: beta-diversity-weighted-unifrac-Sex-Diet-significance_permanova-pairwise; BioSampleObjects; BioSampleObjectsURLs
